# Influence of Resveratrol on Oxidation Processes and Lipid Phase Characteristics in Damaged Somatic Nerves

**DOI:** 10.1155/2019/2381907

**Published:** 2019-12-11

**Authors:** S. I. Pinyaev, T. P. Kuzmenko, N. V. Revina, M. V. Parchaykina, A. S. Pronin, I. V. Syusin, O. S. Novozhilova, V. V. Revin, E. V. Chudaikina, E. S. Revina

**Affiliations:** Department of Biotechnology, Bioengineering and Biochemistry, National Research Ogarev Mordovia State University, Saransk, Russia

## Abstract

It has been shown that the intensification of oxidative processes is observed when somatic nerves of rats are damaged. Accumulation of malondialdehyde occurs, and the phase properties of the lipid bilayer change, especially in the distal part of the nerve. Under the same conditions, there are multidirectional changes in the activity of antioxidant enzymes, superoxide dismutase (SOD) activity decreases, and catalase (CAT) activity increases. Under the action of resveratrol, there is a decrease in the number of TBA-active products in both areas of the damaged nerve. Alongside resveratrol action, SOD and CAT activity tends to return towards the control values. Similar patterns are observed in the action of resveratrol on the phase states of lipids with the damage to somatic nerves. By summarizing the data obtained, it can be claimed that when the nerve is damaged, profound changes occur both in the lipid component and in the antioxidant system. Resveratrol has a stabilizing effect on the studied parameters, and a longer period of time is required for their complete recovery.

## 1. Introduction

One of the most pressing problems in neurophysiology and regenerative medicine is the restoration of damaged somatic nerves. The difficulty in solving this problem lies in the disruption of the biochemical and physicochemical processes of the cell at almost all levels of organization, as well as interference with the systems regulating intercellular interactions [1]. Following peripheral nerve injury, oxidative damage can occur owing to increased formation of reactive oxygen intermediates (ROIs) and lipid-derived free radicals, which not only interfere with biochemical reactions but also cause structural nerve damage. ROIs can attack cellular proteins and DNA [2], alter tubulin formation [3], and disrupt synaptic transmission and ion channel functions [4]. These processes are referred to as oxidative stress, which is one of the main causes of nerve damage [5].

The promotion of oxidative processes disrupts the mechanism of cellular ion transport [6] and leads to sharp decreases in the Na^+^, K^+^, and Ca^2+^ gradients [7]. All effects associated with the disturbance of both passive and active ion-transporting systems are mediated by the increased lipid peroxidation (LPO) in the lipid phase [7–9].

The accumulation of significant amounts of oxidation products in lipids alters the phase characteristics of the lipid bilayer [10], which can affect various membrane-associated enzyme systems [11].

It is known that the accumulation of LPO products in myelin leads to changes in the activity of ion-transporting systems, altered protein conformations, the appearance of nonselective ion channels, and a change in microviscosity, which disrupt the normal functioning of the nerve fiber, as well as changes in the individual composition of phospholipids and fatty acids, which alter the phase characteristics of the membrane [12, 13].

To date, numerous physiologically active compounds that exert simultaneous effects on both LPO processes and regulation of the synthesis of various enzyme systems, including antioxidant systems, via modulating the signaling pathways have been reported [14].

One main goal of our research is to search for physiologically active compounds that not only have antioxidant effects or enhance the activity and synthesis of antioxidant enzymes but also enhance regeneration processes in somatic nerves.

To date, a number of physiologically active compounds are known, which have a simultaneous effect on the processes of LPO by the help of three mechanisms: direct inactivation of free radicals [15], activation of antioxidant enzymes [16], and induction of antioxidant enzyme expression through a mechanism involving signaling pathways [17].

A large number of natural compounds, both of flavonoid nature and of other classes that have neurotrophic properties, influence the neuron proliferation, survival, growth, and differentiation and are widely discussed and investigated in the literature. A number of polyphenols, including flavonoids such as baicalein, daidzein, and luteolin, as well as nonflavonoid polyphenols such as aurapten, carnosic acid, and curcuminoids, increase neuron survival and facilitate neuron growth *in vitro* [14].

One such promising natural compound is resveratrol (3,5,4′-trihydroxystilbene), which consists of two phenolic rings connected via a *trans* double bond and exhibits antioxidant activity [18].

It is able to cross the hematoencephalic barrier and, accordingly, can be localized in brain tissue and have neuroprotective and neuromodulation effects in various pathologies of the brain [19].

It is also reported that resveratrol, along with other flavonoids, can increase the amount of BDNF [20]. Resveratrol has a neurotrophic effect on dopaminergic neurons [21], and an antidepressant-like effect was also found in rats by activating the CREB signaling pathway in the hippocampus and amygdala [22].

It also has its neuroprotective effect by activating the HIF-1 pathway and antioxidant and antiapoptotic properties by activating antioxidant enzymes [16].

Based on this goal, in this work, we addressed several outstanding questions. First, we assessed the level of oxidative processes in damaged somatic nerves. Second, we investigated the influence of these oxidative processes on the phase properties, which are responsible for the state of numerous protein structures localized in the bilayer. Third, we identified the composition and role of antioxidant enzymes in nerve damage, using SOD and CAT as representative examples. Fourth, we examined the possible application of resveratrol as a promising physiologically active compound for the regeneration of somatic nerves.

## 2. Materials and Methods

**Figure 1:**
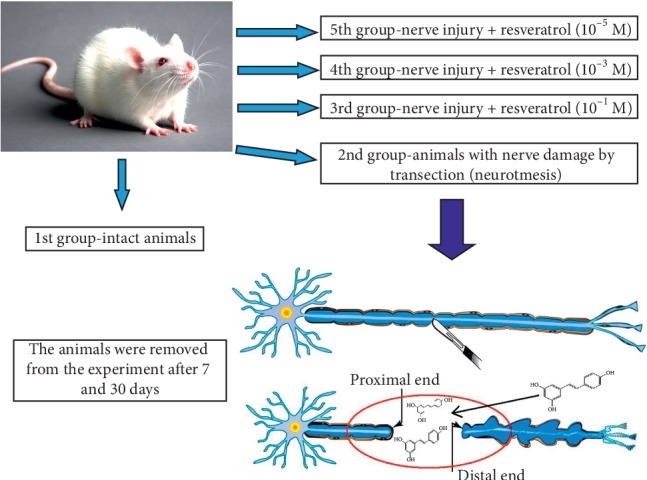
Experiment design.

The object of the study was white male rats of the Wistar line, 24–30 weeks old, weighing 200–250 g. The focus of this study was the sciatic nerve. The rats were kept under vivarium conditions with a 12 h light/12 h dark cycle and free access to food and water. The animals were divided into five groups (Figure 1).

The first group comprised animals without sciatic nerve damage (intact). The second group comprised animals subjected to neurotmesis (cutting) of the sciatic nerve. After shaving and treatment of the working area with 70% ethyl alcohol, a vertical incision was made. The left sciatic nerve, located after dissection of the gluteus maximus muscle, was dissected to the proximal and distal ends. The wound was then sutured in a layer-by-layer manner, and paralysis of the left foot and shin was considered to indicate successful simulation of the injury. All operations were performed by a single researcher, and nerve dissection was performed at the level of the middle third of the thigh.

Animals belonging to the third, fourth, and fifth experimental groups were subjected to sciatic nerve transection and then injected daily with 100 mcl resveratrol (Shaanxi Honghao Bio-Tech Inc., China) into the broad medial thigh muscle at concentrations of 10^−1^, 10^−3^, and 10^−5^ M, respectively. The animals were removed from the experiment after 7 or 30 d, and the concentration of thiobarbituric acid- (TBA-) active products, SOD and CAT activities, and the phase state of lipids released from the sciatic nerve were examined.

The nerve tissue was homogenized in cold phosphate buffer (pH 7.4), and the resulting homogenate was centrifuged at 10000 ×*g* for 15 min at 4°C (Sorvall RC 6 Plus, Thermo Scientific). The supernatant was used in the subsequent measurements [23].

The concentration of TBA-active products (malondialdehyde) was determined using the method reported by Mihara and Uchiyama [24]. The principle of the method is based on the interaction between MDA and thiobarbituric acid (TBA) in an acidic medium at high temperature to form a colored trimethine complex having a maximum absorption in the region of 532 nm.

The SOD activity was determined by measuring the decrease in the reduction rate of nitro blue tetrazolium in the xanthine/xanthine oxidase system at 550 nm (25°C). [25]. Xanthine in the presence of O_2_ decays to form urate, and the O_2_^−^ reaction is catalyzed by the xanthine oxidase enzyme. The formation of a superoxide anion can be registered by changing the coloring of the NTB dye (nitro blue tetrazolium). NTB is restored to formazan. The reaction is carried out within 2 minutes, and the NTB recovery rate is calculated on a linear section.

The CAT activity, utilizing H_2_O_2_ to form water and oxygen, was determined in the reaction with hydrogen peroxide to reduce the absorption value at 240 nm in nervous tissue homogenate [26].

The total protein content was estimated via the Lowry method [27]. The method is based on the formation of colored products of aromatic amino acids with Folin's reagent in combination with biuret reaction to peptide bonds. The color intensity of the complex, which is proportional to the amount of protein in the test sample, is measured spectrophotometrically.

All measurements were conducted using a spectrophotometer (UV-3600, Shimadzu, Japan).

The phase transition temperature of the lipids was determined using a differential scanning calorimeter (DSC822e, Mettler Toledo, Switzerland). The method of investigation of physical and chemical processes is based on the measurement of thermal effects, accompanying the transformation of substances at a given temperature. Aluminum crucibles with lids were used for the measurement. The test sample was placed in one of the crucibles, and the other crucible (the empty one) was used as a reference one. Measurements were carried out at a heating rate of 3°C per minute in the temperature range from −60 to 60°C in the nitrogen current.

For statistical data processing, MS Excel 2010 spreadsheet programme and Statistica 10 application software package were used. The experiments were carried out in tenfold repeats. The results were analyzed using Student's *t*-test. The values were considered significantly different at *p* ≤ 0.05.

All animals for this study were placed in the Animal Care Facility of Ogarev Mordovia State University under standard conditions with free access to water and food. All animal procedures were performed according to a protocol approved by the Institutional Animal Care and Use Committees of the Medical Institute of Ogarev Mordovia State University (Ethics Committee protocol No. 50 from 20.05.2017).

Experiments involving animals were conducted using the principles of humanity and in accordance with the requirements of the Geneva Convention “International Guiding Principles for Biomedical Research Involving Animals” (1990).

## 3. Results and Discussion

The promotion of LPO processes appears to be of particular importance to the development and course of degenerative processes of peripheral nerves. The main indicator for assessing the occurrence of oxidative stress is the accumulation of metabolites originating from free radical oxidation, including the final product MDA, which undergoes reaction with thiobarbituric acid and is a common indicator used to monitor oxidative stress.

**Figure 2:**
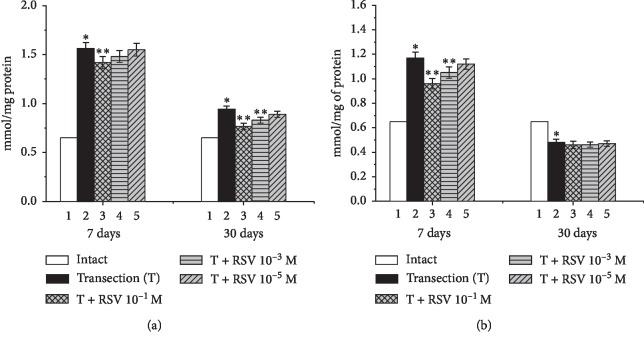
Changes in the levels of TBA-active products in the proximal (a) and distal (b) ends of the damaged sciatic nerve and the influence of various concentrations of resveratrol. ^*∗*^indicates a significant difference relative to the intact group (*p* < 0.05), and ^*∗∗*^indicates a significant difference relative to the untreated group (*p* < 0.05).

The levels of TBA-active products (MDA) in the proximal and distal ends of the damaged nerve were analyzed. It was found that the level of MDA in the first (intact) group of animals was equal to 0.65 mmol/mg of protein (Figure 2).

Seven days after the nerve injury, the MDA levels in the proximal and distal ends of the damaged nerve in the second group had increased by 140% and 80.8%, respectively, compared with the intact group (Figure 2). In the next series of experiments, we administered resveratrol to the animals with a severed nerve. When resveratrol was administered at a concentration of 10^−5^ M, no statistically significant change was observed in the proximal end of the nerve. Upon increasing the resveratrol concentration to 10^−3^ or 10^−1^ M, the amount of MDA decreased by 11.6% and 21.7%, respectively, compared with the untreated group. Similar decreases in the amount of MDA by 8%, 18.8%, and 32.2%, respectively, were observed in the distal end of the damaged nerve after 7 d, compared with the untreated group.

Thirty days after the nerve injury, the MDA levels in the proximal and distal ends in the second group had increased by 45% and decreased by 26.2%, respectively, compared with the intact group (Figure 2). In the experimental groups administered resveratrol concentrations of 10^−1^, 10^−3^, and 10^−5^ M, the MDA levels at the proximal end had decreased by 27.1%, 16.4%, and 8%, respectively, compared with the untreated group. No statistically significant changes were observed at the distal end following resveratrol administration.

Thus, we can conclude that peripheral nerve injury leads to the accumulation of MDA. On the basis of the obtained results, it can be argued that resveratrol inhibits LPO processes. There is also evidence in the literature to suggest that resveratrol suppresses the development of oxidative stress, thereby protecting cell membranes [28, 29].

**Figure 3:**
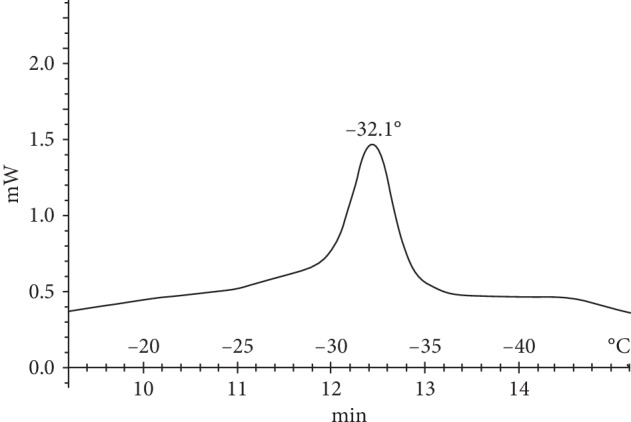
Thermogram of lipids released from the sciatic nerve of the intact group.

**Figure 4:**
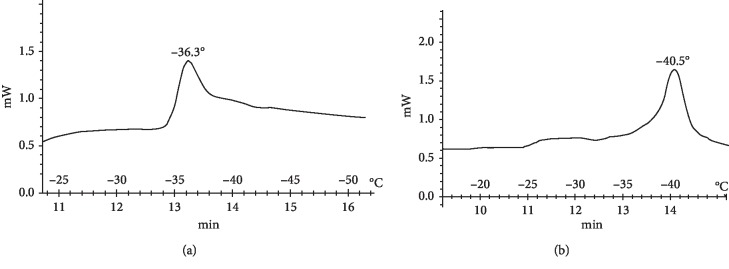
Thermograms of lipids released from the (a) proximal and (b) distal segments of the damaged sciatic nerve after 7 d.

Formed as intermediate products from lipid peroxidation, hydroperoxides are highly hydrophilic and they effectively destroy the bilayer, increasing the availability of the hydrophobic membrane domains for water molecules, and this process of membrane hydration leads to modification of its phase characteristics. On this basis, we expected that the LPO products should lead to a change in the phase state of the nerve fiber membrane. Therefore, we next conducted differential scanning calorimetry experiments using the lipids released from the intact sciatic nerve, which revealed a phase transition temperature of −32.1°C (Figure 3). Seven days after the nerve injury, the lipid phase transition temperature had decreased to −36.3°C in the proximal segment and −40.5°C in the distal segment (Figures 4(a) and 4(b)).

**Figure 5:**
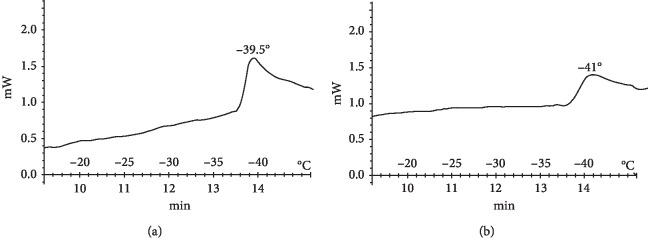
Thermograms of lipids released from the (a) proximal and (b) distal segments of the damaged sciatic nerve after 7 d with daily administration of resveratrol at a concentration of 10^−1^ M.

Following the administration of resveratrol at a concentration of 10^−1^ M for 7 d, further decreases in the lipid phase transition temperature to −39.5°C in the proximal segment and −41°C in the distal segment were observed (Figure 5).

**Figure 6:**
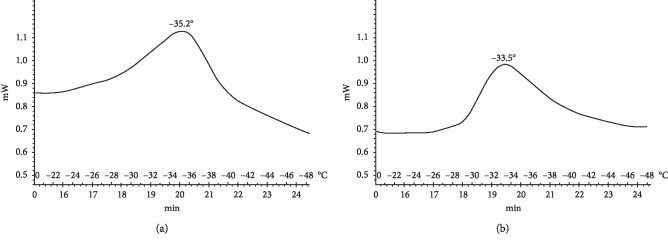
Thermograms of lipids released from the proximal segment of the damaged sciatic nerve after 30 d for (a) the untreated group and (b) the group administered 10^−1^ M resveratrol daily.

Thirty days after nerve transection, the lipid phase transition temperature in the proximal region was −35.2°C for the untreated group. With the daily administration of 10^−1^ M resveratrol, the lipid phase transition temperature in the proximal end was −33.5°C, which is similar to but slightly lower than that observed for the intact group (Figure 6).

Nerve damage therefore contributes to changes in the physicochemical state of the bilayer, leading to a decrease in the lipid phase transition temperature, whereas resveratrol administration was accompanied by an increase in the phase transition temperature. Stilbenes mainly interact with the polar groups of lipids, and some derivatives have been reported to penetrate into the inner regions of the membrane [30]. It is also known from the literature that resveratrol can penetrate into the cell. Although there is evidence that resveratrol can interact with the charged phospholipid groups on the membrane surface, over 90% penetrates into the bilayer space [31] and interacts with nonpolar acyl chains [32], thus affecting the lipid phase transition Changes in the phase state of the membrane bilayer can lead to changes in the activity of membrane-bound enzymes and the state of ion channels and membrane-bound enzymes. On this basis, it can be concluded that changes in the phase state of membrane lipids following damage and the action of resveratrol exert a direct influence on membrane-localized ion-transporting and receptor systems [33, 34].

The level of ROI formation directly influences the activity of the cellular antioxidant system: following a moderate increase in ROIs, activation of the enzyme link of the antioxidant system generally occurs, whereas an excessive increase in the level of free radicals often leads to suppression of the enzymic link of radical cell protection [35]. One of the enzymes of the cellular antioxidant system is SOD. Based on the fact that injury alters the local oxygen supply to a nerve, we next measured the SOD activity in each of the segments of the damaged sciatic nerve. SOD is the first line of defense among antioxidant enzymes and acts to prevent superoxide accumulation and reduce NO-mediated neurotoxicity [36]. The low SOD activity in damaged neurons leads to excessive oxidative damage. Thus, increased SOD activity is crucial for protecting neurons from oxidative damage.

**Figure 7:**
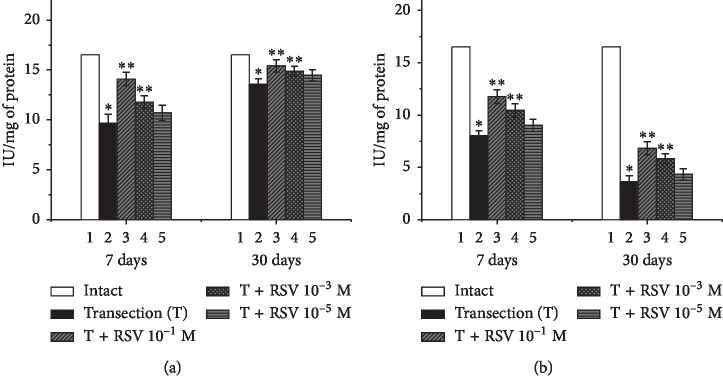
Changes in the SOD activity in the proximal (a) and distal (b) ends of the damaged sciatic nerve and the influence of various concentrations of resveratrol. ^*∗*^indicates a significant difference relative to the intact group (*p* < 0.05), and ^*∗∗*^indicates a significant difference relative to the untreated group (*p* < 0.05).

The results revealed that, in the intact group, the SOD activity was 16.52 IU/mg of protein. Seven days after the nerve damage, the SOD activity in the proximal and distal ends of the nerve had decreased by 41.4% and 51.5%, respectively, compared with the intact group (Figure 7). The daily administration of resveratrol led to partial recovery of the SOD activity in both the proximal and distal ends, compared with the untreated group. The most effective concentration of resveratrol was found to be 10^−1^ M, which increased the SOD activity in the proximal and distal ends by 26.7% and 22.6%, respectively, compared with the untreated group (Figure 7).

Thirty days after the nerve damage, the SOD activity in the proximal and distal ends had decreased by 18% and 78%, respectively, compared with the intact group (Figure 7). The administration of resveratrol at concentrations of 10^−1^, 10^−3^, and 10^−5^ M tended to restore the SOD activity in the proximal end of the nerve; the maximum increase of 11.2% compared to the untreated group was observed for the resveratrol concentration of 10^−1^ M. No significant difference was observed in the distal end of the damaged nerve 30 d after injury upon daily administration of 10^−5^ M resveratrol. However, upon increasing the resveratrol concentration to 10^−3^ or 10^−1^ M, the SOD activity increased by 13.4% and 19.2%, respectively, compared with the untreated group.

Resveratrol is an excellent acceptor of hydroxyl, superoxide, and other free radicals [37], protecting cell membranes from lipid peroxidation and preventing DNA damage caused by the formation of ROIs. The protective effect of resveratrol has been demonstrated to originate from its action on nuclear factor 2 (Nrf2), which is responsible for the elimination of ROIs [38] by activating antioxidant enzymes, such as SOD, CAT, glutathione reductase, glutathione peroxidase, glutathione transferase, and glutathione [39].

The use of natural or synthetic antioxidants holds great promise for the prevention or treatment of neurological disorders. Such compounds facilitate the removal of ROIs and impart resistance to oxidative stress. Resveratrol has been demonstrated to possess considerable antioxidant and anti-inflammatory properties and act as a neuroprotective agent against excitotoxicity [40]. Furthermore, there is evidence in the literature to support not only the direct action of resveratrol as an antioxidant but also its indirect action. Previous studies have revealed that resveratrol can reduce the level of oxidative stress in cells by activating genes responsible for the synthesis of antioxidant enzymes affecting the phosphatase PTEN [41], which in turn increases [42]. This indicates that the PTEN/Akt signaling pathway is involved in the antioxidant effects of resveratrol.

The next step to study the influence of resveratrol on sciatic nerve damage was to investigate the CAT activity, as this enzyme utilizes the hydrogen peroxide formed by SOD. The CAT activity in the intact group was found to be 11.5 IU/mg of protein.

**Figure 8:**
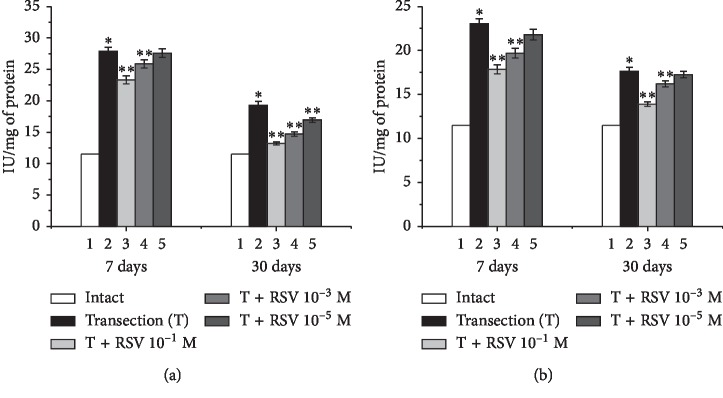
Changes in the CAT activity in the proximal (a) and distal (b) ends of the damaged sciatic nerve and the influence of various concentrations of resveratrol. ^*∗*^indicates a significant difference relative to the intact group (*p* < 0.05), and ^*∗∗*^indicates a significant difference relative to the untreated group (*p* < 0.05).

Seven days after the nerve damage, the CAT activity in the proximal and distal ends had increased by 143% and 100.3%, respectively, compared with the intact group (Figure 8). The daily administration of resveratrol at concentrations of 10^−1^, 10^−3^, and 10^−5^ M decreased the CAT activity in the proximal end by 40%, 18%, and 4%, respectively, and that in the distal end by 45%, 29%, and 10%, respectively, compared with the untreated group (Figure 8).

Thirty days after the nerve damage, the CAT activity in the proximal and distal ends had increased by 68% and 53%, respectively, compared with the intact group. The daily administration of resveratrol at concentrations of 10^−1^, 10^−3^, and 10^−5^ M decreased the CAT activity in the proximal end by 53%, 40%, and 21%, respectively, compared with the untreated group. In the distal end, decreases of 32% and 12% were observed using resveratrol concentrations of 10^−1^ and 10^−3^ M, respectively, whereas the resveratrol concentration of 10^−5^ M did not lead to a significant difference in CAT activity, compared with the untreated group.

The obtained data indicate that nerve damage increases CAT activity and decreases SOD activity. The increase in CAT activity relative to the intact group in all cases is in contrast to the significant decrease in SOD activity. These results are in accordance with literature data, where the phenomenon of cross-regulated activity has been observed for numerous enzymes, including SOD and CAT [43]. In the case of CAT, the superoxide anion radical is a negative effector and H_2_O_2_ is a positive effector, whereas the opposite is true for SOD.

The administration of resveratrol was found to decrease the CAT activity following nerve injury. Recent studies have revealed that CAT transcription is mediated by redox-sensitive transcription factors such as Nrf2 and nuclear factor-kappa B (NFkB), which can influence the redox status after pathophysiological changes [44].

The depletion of cellular antioxidant defense mechanisms and the generation of free radicals lead to the activation of NFkB [45], an inducible transcription factor, thus increasing the expression of genes regulated by NFkB and leading to the increased synthesis of proinflammatory cytokines and protection from oxidative stress [46]. This organized regulation of Nrf2 and NFkB gene expression is very effective for increasing cellular detoxification and antioxidant capacity, demonstrating the important role of NFkB-Nrf2-ARE (antioxidant response elements) pathways as a cellular antioxidant defense system, and resveratrol can regulate the activity of the NFkB pathway [47]. Owing to its polyphenol structure, we can conclude that resveratrol possesses antioxidant properties, which have been predominantly studied *in vitro*. Alongside other polyphenols, resveratrol is of interest owing to its useful properties under various pathological conditions including neurodegeneration [48, 49] and it has been shown to be quite effective for reducing the effects of oxidative stress, thus helping to prevent oxidative damage to cell membranes and organelles.

## 4. Conclusion

There through summarizing the data obtained, it can be claimed that nerve damage leads to profound changes in both the lipid component and the antioxidant system of somatic nerves. Perhaps, the trigger mechanism of many disruptions is the activation of oxidative processes, evidence of which is the accumulation of the final products of lipid peroxidation—malondialdehyde. The source of malondialdehyde formation is unsaturated fatty acids that form a bilayer, and their oxidation uniquely influences the phase state of lipids, which we register. Namely, when the nerve is cut, a sharp shift in the temperature of the phase transition occurs. It is also known that the functional characteristics of a large number of associated enzymes, ion-transporting systems, and receptors depend on the phase transition [33, 34]. We use resveratrol, which is a multifunctional compound, namely, having antioxidant properties [50], an activator of antioxidant enzymes [16]. It also stimulates the synthesis of antioxidant enzymes [17], facilitates the regeneration of damaged nerves, and contributes to the suppression of oxidative processes, which we observed in the experiment. As well as that, we have ascertained its effect on antioxidant enzyme systems and the phase state of the lipid bilayer. Under the action of resveratrol, there is a tendency to restore the disturbed characteristics of somatic nerves, and especially in the part of erethic formations with which the central innervating is preserved. It should also be noted that a more complete recovery requires a longer period of time.

## Data Availability

The data used to support the findings of this study are included within the article.
